# Behavioral and Event-Related Potential Study of Emotion Concept Activation in Young Adults with High Versus Low Alexithymia Traits

**DOI:** 10.3390/brainsci16030264

**Published:** 2026-02-26

**Authors:** Jiafeng Jia, Minggang Zhang, Xiaoying He, Zeming Chen, Xiaochun Wang

**Affiliations:** School of Psychology, Shanghai University of Sport, Shanghai 200438, China

**Keywords:** alexithymia, emotion concept, emotional constructivism, emotion recognition, ERP

## Abstract

**Highlights:**

**What are the main findings?**

- Clear emotion concepts improved facial expression identification accuracy in both high- and low-alexithymia groups.
- The high-alexithymia group showed reduced N400 amplitudes under top-down processing conditions, indicating impaired deliberate activation of emotion concepts.

**What are the implications of the main findings?**

- This study provides direct electrophysiological evidence linking alexithymia to a deficit in the top-down conceptual processing of emotions.
- The findings suggest that therapeutic interventions targeting emotion concept activation could be beneficial for individuals with alexithymia and related affective disorders.

**Abstract:**

**Background**: Although alexithymia is characterized by difficulties in emotional processing, the underlying mechanisms remain uncertain. We hypothesized that specific deficits in activating and using emotion concepts would be associated with impairments in higher-order emotional processing in individuals with high levels of alexithymia. **Methods**: To elucidate these mechanisms, 20 high-alexithymia and 17 low-alexithymia young adults (M_age_ = 18.38, SD_age_ = 0.77), identified according to the Toronto Alexithymia Scale-20, were included in this study to examine distinct neural and behavioral features between participants with different levels of alexithymia. Participants selected target facial expressions primed by emotion concepts from interferential faces while their event-related potentials (ERPs) were recorded. We modulated the clarity of emotion concepts and varied the relative working-memory load of the emotion concepts versus facial features to promote top-down or bottom-up processing. **Results**: Behaviorally, clear emotion concepts facilitated accurate target identification in both groups. Event-related potential results show that the high alexithymia group had reduced N400 amplitudes than the low-alexithymia group in the top-down domain processing condition (mean difference of 2.75 μV, 95% CI [0.40, 5.11], *Cohen’s d* = 0.54), indicating reduced cognitive resource allocation for deliberately activating emotion concepts. **Conclusions**: These findings suggest that individuals with high alexithymia have emotion deficits, potentially due to difficulty in the deliberate activation of emotion concepts. Our findings provide theoretical and clinical implications for affective science by highlighting a possible conceptual-processing mechanism through which alexithymia may be linked to the development and persistence of comorbid affective symptoms.

## 1. Introduction

Individuals with alexithymia experience difficulty identifying and describing feelings, possess a concrete, reality-based cognitive style, and exhibit impoverished inner emotional and fantasy lives [1]. Approximately 10% of the general population exhibits symptoms of alexithymia [2]. Alexithymia is associated with multiple physical and psychological disorders, including non-suicidal self-injury (NSSI) [3], depression and anxiety [4,5,6], substance abuse [7,8], and internet addiction [9]. Social maladjustment is also associated with alexithymia, including social indifference [10] and social withdrawal [11]. Although alexithymia has been linked to difficulties in emotional processing, converging neuroimaging and neurophysiological findings now point to altered activity and connectivity within limbic–prefrontal and salience-network regions (e.g., amygdala, insula, cingulate cortex, and prefrontal areas) as putative mechanisms underlying this association [12,13,14]. Meta-analytic work shows reduced responses to negative stimuli in the amygdala and dorsomedial prefrontal cortex, and to positive stimuli in the right insula and precuneus, alongside increased dorsal anterior cingulate activity, pointing to atypical affective weighting and compensatory cognitive control during emotion processing [15]. Structural meta-analysis further reveals reduced gray matter volume in the left insula, left amygdala, orbitofrontal cortex, and striatum—regions critical for emotion perception, experience, and reward-based learning [16]. Consistent with these findings, recent resting-state work has documented decreased clustering and modularity within social–semantic hubs such as the left posterior middle temporal gyrus in alexithymia, interpreted as less efficient and less specialized emotion–semantic processing [17]. Despite these important contributions, the specific cognitive and neural mechanisms that underlie altered emotion processing in alexithymia—particularly the role of emotion concept activation—remain insufficiently understood. Emotional constructivism offers a new perspective on the nature of emotions and may explain alexithymia.

Emotional constructivism posits that emotions are not inherent or universally fixed [18,19] but, rather, are individually constructed through experience, culture, and social context [20]. Emotions are not distinct mental states; instead, they emerge from an ongoing, continually evolving process intertwined with cognition and perception [21,22]. In this framework, emotions are viewed as being psychologically constructed through a process referred to as situated conceptualization [23], meaning that individuals conceptualize emotions when making sense of situations and core affect (a neurophysiological state consciously accessible as a simple, non-reflective feeling that is an integral blend of hedonic and arousal values) [24]. Emotion concepts play a crucial role in this process.

Emotion concepts” are defined as abstract knowledge structures for emotion categories (e.g., “anger,” “sadness”) that encode typical causes, bodily sensations, expressions, and action tendencies [25,26,27,28]. These concepts provide the semantic basis for making sense of affective states in context and are gradually constructed as learners encounter variable emotional episodes paired with emotion words. This differs from “emotion recognition”, the online process of identifying an emotion from cues such as facial or vocal expressions in a specific instance; from “emotion labeling”, the act of assigning a verbal label to one’s own or others’ states; and from “emotional granularity”, which captures individual differences in how precisely people distinguish and report their emotional experiences using fine-grained categories [29,30]. In this framework, emotion concepts constitute the underlying knowledge system that supports both recognition and labeling. The language hypothesis also proposes that alexithymia stems from a language impairment that prevents the formation of discrete emotion concepts from ambiguous affective states, thereby compromising the ability to identify and describe feelings [31,32]. For example, when the semantic satiation task was used to reduce participants’ access to emotion concepts, their ability to correctly judge whether two facial expressions represented the same emotion was significantly impaired [33]. Moreover, individuals with language dysfunction exhibit impairments in emotional perception. For example, individuals with semantic aphasia show deficits in recognizing facial emotions anchored by emotion words without expressive cues [34]. Patients with primary progressive aphasia demonstrate impairments in both emotion concept knowledge and emotion recognition. Studies have also shown that varying the words or phrases used to describe emotional experiences can influence how individuals understand and communicate their feelings [35,36,37]. Furthermore, researchers have coined the term “emotional granularity” to refer to the number of emotion concepts one has learned [38]. Individuals with higher emotional granularity have higher life satisfaction, better interpersonal relationships, and lower levels of anxiety, depression, and other symptoms of mental disorders [29,39]. Together, these findings suggest that impaired activation of emotion concepts may contribute to the emotional processing deficits present in individuals with alexithymia.

Emotion concepts help transform ambiguous affect (e.g., unpleasant feelings) into specific, discrete emotions (e.g., anger). However, how emotion concepts function in individuals with alexithymia remains unexplored.

The N400 is a negative-going ERP component peaking around 400 ms that is classically elicited by meaningful stimuli such as words, pictures, or faces in context, with larger amplitudes for items that are harder to integrate with prior semantic or emotional information [40,41]. Beyond language, N400 effects have been reported for emotional and conceptual mismatches across modalities, including faces and emojis paired with incongruent contextual cues [42,43]. Prior research has also reported N400 in recognition memory tasks and in decision phases where previously encoded semantic or contextual information is re-activated and integrated [44,45] These findings support the view that the N400 is not restricted to verbal semantics but indexes context-sensitive construction of emotional and conceptual meaning across different modalities. The N400 thus provides a robust index of context-dependent semantic integration, making it particularly well suited for examining the activation and construction of emotion concepts.

Collectively, emotion concepts constitute a fundamental component of emotional processing. However, their role has been relatively neglected in alexithymia research, and the precise mechanisms through which they influence emotional processing in individuals with alexithymia remain elusive. Prior ERP studies on alexithymia have documented both early perceptual–attentional anomalies and later regulation difficulties. Specifically, individuals with alexithymia show reduced automatic attention to emotional cues and blunted early visual responses (e.g., P1/N170) to faces, reflecting weak initial encoding of affective information [46,47,48]. At the later stage, control and monitoring processes—indexed by the P3 and late positive potentials (LPP)—appear less efficient [49,50]. However, the intermediate process of constructing emotion through emotion concepts remains insufficiently studied.

To address this gap, the present study combined behavioral paradigms with event-related potential (ERP) recordings in a sample of young adults to examine the roles of emotion concepts in emotional processing among individuals with high alexithymia and to characterize the distinctive patterns that differentiate them from individuals with low alexithymia. We hypothesized that, compared to low-alexithymia individuals, high-alexithymia individuals would exhibit poorer behavioral performance on tasks that rely on the activation and integration of emotion concepts; at the neural level, we expected altered N400 to emotion-concept-incongruent conditions indexing less efficient activation and integration of emotion concepts.

## 2. Methods

### 2.1. Participants

We initially recruited 701 first-year university students who completed the Chinese version of the 20-Item Toronto Alexithymia Scale (TAS-20) [51,52]. Following standard cut-offs used in the Chinese literature [53,54], individuals with TAS-20 scores ≥ 61 were classified into the high-alexithymia (HA) group, and candidates for the low-alexithymia (LA) group were selected from those with the lowest TAS-20 scores up to 51. Alexithymia is known to be positively associated with depression and anxiety symptoms [55,56,57]. To reduce potential confounding effects of depression and anxiety, all 701 students also completed the Chinese versions of the Center for Epidemiological Studies Depression Scale (CES-D) [58] and the Generalized Anxiety Disorder 7-Item Questionnaire (GAD-7) [59], and only those without clinically relevant depressive or anxiety symptoms (GAD-7 < 10, CES-D < 16) were eligible for the ERP experiment. This led to a substantial reduction in the pool of eligible participants but allowed us to obtain two groups with comparable gender distribution (HA, 10 female; LA, 9 female).

Based on a medium effect size reported in previous alexithymia studies (η^2^ ≈ 0.06) [47,49] and a priori power analysis (α = 0.05, *η_p_*^2^ = 0.80), at least 28 participants were required. We therefore included 37 participants in the final sample, exceeding the minimum requirement: 20 HA (mean TAS-20 = 67.60, range = 61–80) and 17 LA (mean TAS-20 = 42.81, range = 20–51; total sample: M_age_ = 18.38, SD_age_ = 0.77; see Table 1).

It is important to acknowledge that alexithymia is a multidimensional construct [60,61,62]. For example, factor-analytic work on the TAS-20 consistently supports three related but separable components—Difficulty Identifying Feelings (DIF), Difficulty Describing Feelings (DDF), and Externally Oriented Thinking (EOT). To ensure adequate reliability of the screening instrument, we examined the psychometric properties of the TAS-20) in the present sample. Internal consistency was acceptable to excellent for DIF (Cronbach’s α = 0.94), DDF (α = 0.78), and the total scale (α = 0.89), but low for EOT (α = 0.51). This pattern is consistent with prior work showing that the EOT subscale often has weaker reliability and problematic item loadings [62,63], particularly in translated and cross-cultural versions of the TAS-20 [61,62,64].

A confirmatory factor analysis (WLSMV estimator for ordered categorical items) tested the standard three-factor structure (DIF, DDF, EOT). Model fit indices were: χ^2^(167) = 293.28, *p* < 0.001, CFI = 0.976, TLI = 0.972, RMSEA = 0.143. Despite the elevated RMSEA, the high CFI and TLI values suggest adequate comparative fit for the intended three-factor solution, in line with broader evidence that the TAS-20 reasonably captures a three-dimensional alexithymia construct across diverse samples [62,65]. Given the extensive prior validation of the TAS-20 and our use of only the total score for grouping participants (high vs. low alexithymia), we judged the scale to be sufficiently reliable for screening purposes [65,66].

### 2.2. Stimuli and Design

**Figure 1:**
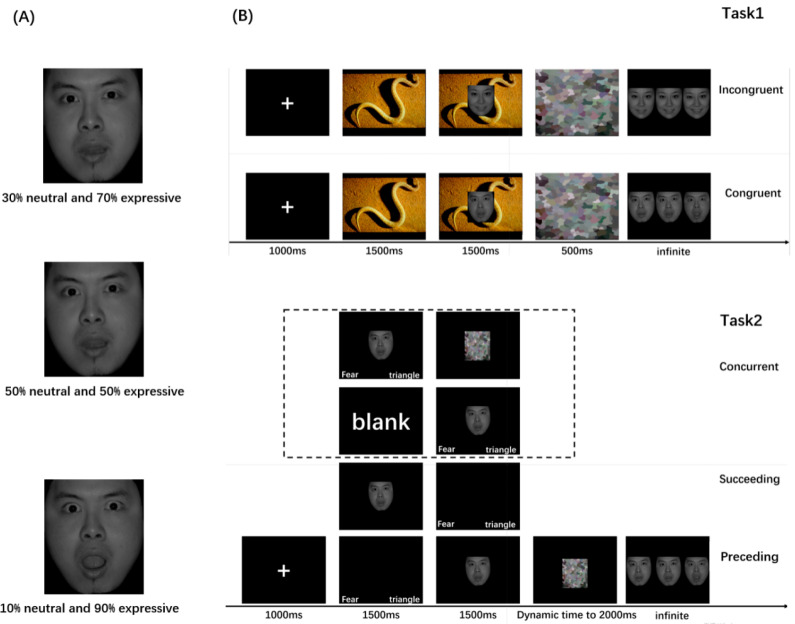
(**A**) An example in which the neutral faces are continuously morphed into expressive faces. (**B**) Procedure of Task 1 and Task 2.

Stimulus faces were selected from the Chinese Facial Affective Picture System (CFAPS) [67,68], with 6 images for each of the five expressions; i.e., happiness, fear, sadness, disgust, and anger (3 male and 3 female faces for each expression). Additionally, neutral expressions of the same faces were used. “Abrosoft FantaMorph 5” was employed to continuously morph the neutral faces into expressive faces, creating images that were 10% neutral and 90% expressive, 30% neutral and 70% expressive, and 50% neutral and 50% expressive (see Figure 1A). The morphed facial expressions were validated in an independent pilot sample of *N* = 30 college students (not included in the ERP experiment). For each identity, the three morphs of the same face were presented as a set. Participants first categorized the emotion of the whole set (forced choice among six emotion labels), and then rated the emotional intensity of each of the three morphed faces on a 7-point Likert scale. Only sets for which at least 80% of participants correctly identified the target emotion, and for which the three morphs showed a consistent ordering of mean intensity ratings from low to high morph level, were retained for the main experiment.

#### 2.2.1. Task 1

Stimuli were displayed by E-Prime 3.0. During a given trial, a fixation point “+” appeared in the center of the screen for 1000 ms. Next, an emotion-laden picture from the International Affective Picture System (IAPS) appeared, followed by a target face, and the picture was covered by a congruent or incongruent context. The target face was presented for 1500 ms, followed by a mask that appeared randomly for 500 to 1000 ms. Subsequently, three faces appeared on the screen, and participants were required to select the target face (see Figure 1B). There were 90 trials in each congruent and incongruent context. Task 1 used a 2 (Group: LA vs. HA) × 2 (Contest: Congruent, Incongruent) mixed factorial design.

#### 2.2.2. Task 2

Task 2 was similar to Task 1, except that it included three types of context manipulations: (1) Preceding Context, in which an emotion word congruent with the target face and a geometric word were presented on a screen for 1500 ms prior to the target face; (2) Concurrent Context, in which the target face was presented simultaneously with the emotion word and geometric word on the same screen; and (3) Succeeding Context, in which after the offset of the target face, an additional screen was presented for 1500 ms displaying the emotion word and geometric word. Participants were required to indicate which word was the emotion word (to ensure that they had processed the words). To ensure that the duration from the target face to the response screen remained constant, a mask screen displayed a dynamic duration (see Figure 1B). There were 90 trials in each Preceding, Concurrent, and Succeeding Context. Task 2 used a 2 (Group: LA vs. HA) × 3 (Contest: Preceding, Concurrent, Succeeding) mixed factorial design.

In both tasks, faces with 30% neutral and 70% emotional intensity served as the target faces. On each trial, participants chose one of three morphed faces: a less intense face (50% neutral/50% emotional), the target face (30% neutral/70% emotional), or a more intense face (10% neutral/90% emotional). We coded each response as a “bias score.” Choosing the more intense face was coded as +1 (e.g., when primed with the 30/70 face but selecting the 10/90 face), choosing the less intense face was coded as −1 (relative underestimation of intensity), and choosing the target face was coded as 0 (no bias). For each participant and condition, we then averaged the trial-wise bias scores to obtain a mean bias score. Negative mean values indicate a tendency to choose faces that are more subtle than the target (bias toward underestimating emotional intensity), whereas positive mean values indicate a tendency to choose more intense faces (bias toward overestimating emotional intensity). The absolute value of the mean bias score (|mean bias|) reflects the magnitude of deviation from the target intensity, with values closer to 0 indicating less bias and thus higher accuracy in that condition.

We employed continuous morphing to transform a neutral face into its expressive counterparts, thereby enabling quantification of facial features (or emotional content) and generating a spectrum of expressions ranging from subtle to more pronounced. The intermediate image from this continuum was selected as the target stimulus (30% neutral and 70% expressive). To investigate how emotion concept clarity influences performance, participants completed tasks with explicit emotion words (Task 2) or ambiguous/conflicting emotion-laden pictures (Task 1). Deviations between their chosen face and the target were used to quantify judgment accuracy and infer selection strategies. To compare the overall choice bias between Task 1 (ambiguous emotion concepts) and Task 2 (clear emotion concepts), an overall bias score was calculated for each task by averaging scores across all participants and experimental conditions within that respective task.

To address the other crucial question regarding how emotion concepts guide judgments—whether they are used for top-down matching or bottom-up confirmation of emotional content—we varied the presentation order of emotion words and faces in Task 2. Specifically, we modulated the relative working memory load of the emotion concepts versus emotion contents. In the Preceding Context, the word preceded the face, giving the facial features greater weight in working memory before choosing the target face, thereby promoting a bottom-up process of face selection. Conversely, in the Succeeding Context, the emotion concepts had greater weight, favoring a top-down process for selecting the target face; the Concurrent Context balanced the contributions of both, thereby allowing us to titrate the relative contribution of top-down matching or bottom-up confirmation.

### 2.3. Electrophysiological Recording and Data Analysis

#### 2.3.1. Electrophysiological Recording

Participants sat in a comfortable chair in a sound-attenuated room with dim lighting, facing a portable computer screen at a distance of 1 m. For EEG recordings, 64 sintered Ag/AgCl scalp electrodes were placed according to the international 10–20 system using an EEG cap. Continuous EEG signals were recorded from a BrainAmp amplifier (Brain Products GmbH, Gilching, Germany) and sampled at 1000 Hz. All data and signal processing were performed using custom-written routines in MATLAB (version R2020b) and the EEGlab plugin (version 2020.0). Offline data were re-referenced to the average of both mastoids (TP9 and TP10) and filtered with a forward 0.1 Hz high-pass filter and a zero-phase 30 Hz low-pass filter. A natural-gradient logistic independent component analysis (ICA) was performed on the data (using the runica algorithm in the EEGLAB toolbox to decompose mixed EEG signals into their underlying neural and artificial components). And artificial components (e.g., ocular artifacts, muscle activity or electrode drift) were excluded from further analysis.

#### 2.3.2. Event-Related Potential

For each participant, average ERP waveforms were computed separately for each condition across trials. Filtered data were segmented from 200 ms before the response-screen stimulus onset until 2000 ms after stimulus presentation. The 200 ms pre-stimulus onset was used for baseline correction. Contaminated epochs were removed via computerized artifact rejection with a peak-to-peak threshold (±100 μV). After preprocessing, we ensured that each condition contained more than 60 valid trials; on average, over 80% of trials per condition were retained.

The electrodes of interest were selected a priori based on the well-established scalp distribution of the N400 component, as supported by prior literature [69,70,71]. Accordingly, the N400 component was quantified as the mean amplitude within the 400–500 ms time window post-stimulus, averaged across the following centro-frontal electrode cluster: F3, Fz, F4, FC3, FC4, C3, Cz, and C4. After confirming that the N400 was indeed observable at these sites in our grand-average waveforms, statistical analyses were then performed on the mean amplitudes extracted from this pre-defined cluster.

#### 2.3.3. Statistical Analysis

To examine the effects of Group and Context on Bias score and N400 amplitude, we fitted a linear mixed-effects model (LMM) using the lmer function in R(version 4.4.1) [72]. The model was specified as:

Measures~Group + Context + Group ×Context + (1|ID).(1)

where Group (HA, LA) and Context (Congruent, Incongruent/Preceding, Concurrent, Succeeding) were entered as fixed factors, and participant (ID) was included as a random intercept to account for individual differences in baseline Bias score and ERP responses.

Given the relatively small sample size (*N* = 37) and the complexity of the variance-covariance structure, we applied the Kenward–Roger approximation to estimate denominator degrees of freedom and adjust standard errors. This method provides more accurate Type I error control for LMMs with small-to-moderate samples compared to traditional approaches [73,74].

Fixed effects were evaluated using Type III analysis of variance with Satterthwaite’s method. Following a significant Group × Context interaction, simple-effects analyses were conducted to compare groups within each context, using *t*-tests with Kenward–Roger adjusted degrees of freedom. Effect sizes for significant comparisons are reported as Cohen’s d, calculated using the pooled standard deviation.

Model selection was guided by likelihood ratio tests comparing nested random-effect structures [75]. The final model retained random intercepts only, as the inclusion of random slopes did not significantly improve model fit and led to convergence issues, consistent with recommendations for modest sample sizes. To assess the robustness of the model, we conducted several additional checks. First, we refitted the models with inclusion of random slopes e.g., Context by participant (1 + Context|ID). These models did not improve model fit according to likelihood ratio tests and resulted in convergence problems, so we retained the more parsimonious random-intercepts-only structure. Second, we verified the normality and homoscedasticity of the residuals; no strong deviations from model assumptions were observed. Third, we refitted the models using restricted maximum likelihood (REML) estimation and obtained essentially identical fixed-effect estimates and significance patterns.

The face images were selected from the Chinese Affective Face Picture System (CAFPS, Wang & Luo, 2005, Gong, Huang, Wang, & Luo, 2011) [67,68], and all the face models in the CAFPS gave their consent for publication for research purposes.

## 3. Results

### 3.1. Task 1

#### 3.1.1. Bias Score

The fixed-effects estimate (β = −0.082, SE = 0.016, t(35) = −4.99, *p* < 0.001) indicated that Context had a significant effect on the bias score. Type III ANOVA confirmed that the main effect of Context was significant (F(1,35) = 24.94, *p* < 0.001, *η_p_*^2^ = 0.42). Specifically, the bias score of incongruent context was lower than congruent context, with M_incongruent_ = −0.001 and M_congruent_ = 0.08 (see Table 2). In contrast, the interaction between Group and Context was not significant (F(1,35) = 0.88, *p* = 0.354). The main effect of Group was also not significant (F(1,35) = 0.22, *p* = 0.643). Although the Group effect was not significant, the accuracy of the LA group was still higher than that of the HA group, with an absolute value of M_LA_ = 0.032 (see Table 2) and an absolute value of M_HA_ = 0.047. The complete descriptive statistics are presented in Table 2. The overall bias score for Task 1 computed across all participants and conditions was 0.080 (M_Task1 bias_ = 0.080, SE = 0.013).

#### 3.1.2. N400 Amplitude

**Figure 2:**
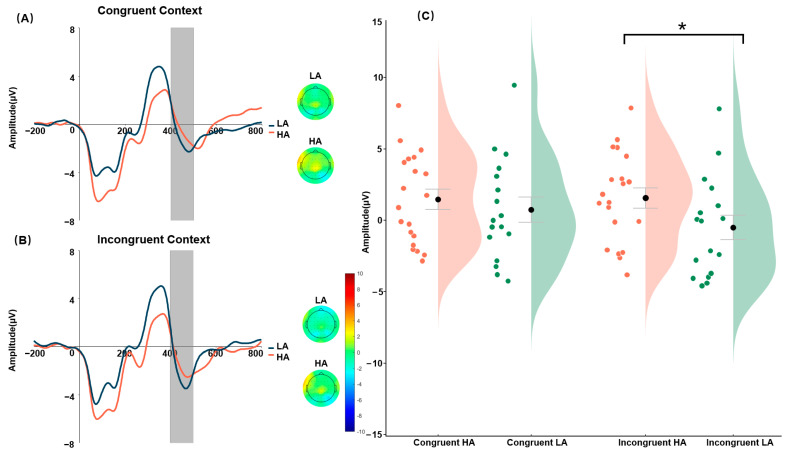
Panels (**A**,**B**) show the grand-average ERP and scalp topographic distributions of Task 1. Panel (**C**) shows the distribution of mean N400 amplitudes across experimental conditions, red represents the high-alexithymia (HA) group and green represents the low-alexithymia (LA) group; dots indicate individual participants’ mean N400 amplitudes and black dots represent the group means. Note: Shaded areas correspond to the time window for the N400 (400–500 ms). * *p* < 0.05.

The fixed-effects estimate (β = 1.566, SE = 0.807, t(35) = −2.29, *p* = 0.028) revealed a significant interaction between Group and Context with regard to N400 amplitude. Type III ANOVA confirmed that the interaction between Group and Context was significant (F(1,35) = 5.26, *p* = 0.028, *η_p_*^2^ = 0.10). Follow-up simple-effects analyses revealed that the mean N400 amplitude within the incongruent context differed significantly between the HA and LA groups (*t*(42.6) = 2.15, *p* = 0.037; *Cohen’s d* = 0.65). Within this context, descriptively, the HA group showed a positive mean amplitude (M = 1.62 μV, SE = 0.59), while the LA group showed a negative mean amplitude (M = −0.61 μV, SE = 1.04). Notably, the LA group’s mean was associated with considerable variability, as indicated by a standard error larger than the absolute value of the mean. In contrast, no significant difference was found between HA and LA in the congruent context (*t*(42.6) = 0.52, *p* = 0.606). The complete descriptive statistics are presented in Table 2 and illustrated in Figure 2.

Note that the degrees of freedom were estimated using the Kenward–Roger approximation, which adjusts for small-sample bias in linear mixed models. This method often yields larger (non-integer) degrees of freedom than traditional approaches, improving the accuracy of statistical inference and Type I error control.

### 3.2. Task 2

#### 3.2.1. Bias Score

The fixed-effects analysis revealed that Context significantly affected the bias scores (β = 0.078, SE = 0.022, t(70) = 3.54, *p* < 0.001). Type III ANOVA confirmed a significant main effect of Context (F(2,70) = 8.99, *p* < 0.001, *η_p_*^2^ = 0.20). Post hoc tests revealed that the Succeeding Context had the lowest bias score, with M_succeeding_ = −0.082; the Preceding Context had the highest bias score, with M_preceding_ = 0.028; and the Concurrent Context had a score of M_concurrent_ = −0.020 (see Table 2). The interaction between Group and Context was not significant (F(2,70) = 0.88, *p* = 0.418), and the main effect of Group was also not significant (F(1,35) = 0.40, *p* = 0.530). Although the Group effect was not significant, the accuracy of LA was still better than that of HA, with an absolute value of M_LA_ = 0.014 and an absolute value of M_HA_ = 0.053. The complete descriptive statistics are presented in Table 2. The overall bias score for Task 2 (computed across all participants and conditions) was −0.025 M_Task2 bias_ = −0.025, SE = 0.016.

#### 3.2.2. N400 Amplitude

**Figure 3:**
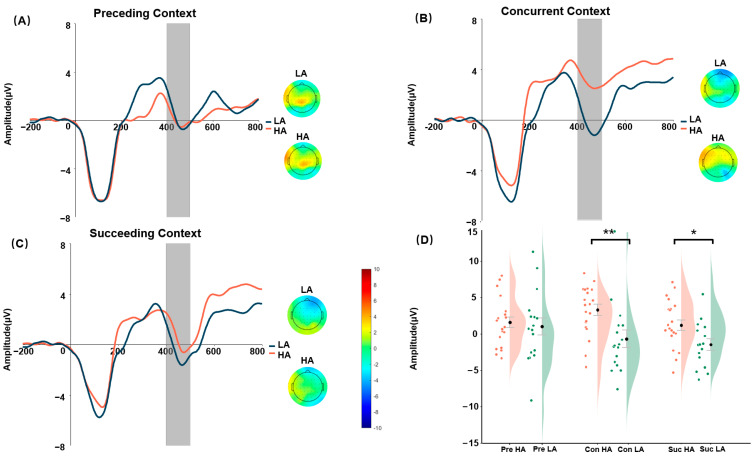
Panels (**A**–**C**) show the grand-average ERPs and scalp topographic distribution of Task 2. Panel (**D**) shows the distribution of mean N400 amplitudes across experimental conditions, red represents the high-alexithymia (HA) group and green represents the low-alexithymia (LA) group; dots indicate individual participants’ mean N400 amplitudes and black dots represent the group means. Note: Shaded areas correspond to the time window for N400 (400–500 ms). Preceding Context of HA (Pre HA), Concurrent Context of HA (Con HA), and Succeeding Context of HA (Suc HA). * *p* < 0.05, ** *p* < 0.01.

The fixed-effects estimate (β = 3.757, SE = 1.375, t(70) = 2.73, *p* = 0.008) revealed a significant interaction between Group and Context with regard to the N400 amplitude. Type III ANOVA confirmed that this interaction was significant (F(2,70) = 3.73, *p* = 0.029, *η_p_*^2^ = 0.10). Follow-up simple-effects analyses revealed that the mean N400 amplitude within the Concurrent Context differed significantly between the HA and LA groups (*t*(62.5) = 3.19, *p* = 0.002; *Cohen’s d* = 0.80) and differed significantly in the Succeeding Context (*t*(62.5) = 2.14, *p* = 0.036; *Cohen’s d* = 0.54). In contrast, no significant difference was found between HA and LA in the Preceding Context. Specifically, the LA group showed significantly more negative N400 amplitudes in the Concurrent Context (M_HA_ = 3.21 μV, SE = 0.89; M_LA_ = −1.43 μV, SE = 1.04) and the Succeeding Context (M_HA_ = 1.02 μV, SE = 0.84; M_LA_ = −1.73 μV, SE = 0.80), but not in the Preceding Context. The complete descriptive statistics are presented in Table 2 and illustrated in Figure 3.

Note that the degrees of freedom were hereafter approximated using the Kenward–Roger method.

## 4. Discussion

In this study, facial expression processing was examined using behavioral and ERP measures to investigate how emotion concepts modulate emotional processing in young adults with alexithymia. We modulated the clarity of emotion concepts (ambiguous/conflicting emotion-laden pictures versus explicit basic emotion words) and the relative working memory load of emotion concepts versus facial features to promote top-down or bottom-up facial expression selection. Facial expression features were quantified to assess selection bias under the influence of different emotion concepts.

A critical difference between Task 1 and Task 2 was the precision of the emotion concept. Task 1 presented an imprecise and ambiguous concept (emotion-laden pictures) of the most likely emotion, whereas Task 2 provided an explicit, verbally grounded emotion concept (basic emotion words). Behavioral results revealed that participants were more accurate in judging the target face in Task 2, which involved explicit emotion concepts, than in Task 1, which involved ambiguous concepts (M_Task1 bias_ = 0.080, M_Task2 bias_ = −0.025). This preference for more subtle faces may indicate that participants were able to make choices based on relatively modest differences in the emotional content of the facial features, which is consistent with a potential facilitatory role of clearer emotion concepts in guiding judgments under ambiguity. At the same time, given the small mean differences in bias scores, this pattern should be interpreted cautiously and may also reflect changes in response strategy rather than a reduced need for perceptual information per se. In this sense, our findings are broadly in line with proposals that variation in the accessibility of emotion concepts can influence facial expression judgments [33,35] but they do not allow for strong causal conclusions about the underlying mechanisms.

Notably, the smallest deviation from the target occurred when the contextual cue was incongruent (M_incongruent_ = −0.001) in Task 1. This finding implies that, when an emotion concept is unhelpful or even disruptive, participants tend to predominantly focus on low-level emotion content (facial features), reflecting a more bottom-up process. Emotion perception is a complex interplay between top-down and bottom-up processes, but it is not clear which process is dominant. To address this issue, we modulated the relative working memory load of emotion concepts versus emotion contents in Task 2 to optimize the weighting of top-down or bottom-up influences. The results from Task 2 revealed a more radical bias in the Succeeding condition (M_succeeding_ = −0.082) than in the Preceding condition (M_preceding_ = 0.028). This pattern indicates that participants were more inclined to use emotion concepts to match and interpret facial features, reflecting more top-down processing. Proponents of emotional constructivism posit that top-down processes are dominant when emotion concepts are incorporated into emotion perception [37,76]. Furthermore, developmental research on children’s emotional understanding suggests that perceptual–predictive power diminishes with age, while the contribution of conceptual knowledge steadily increases. This shift indicates that emotional understanding is a gradually constructed process shaped by the accumulation of experience and knowledge [77]. Behavioral results from Task 2 suggest that in both the LA and HA groups, clear and explicit emotion concepts facilitated recognizing facial expressions, and individuals tended to use emotion concepts to match and interpret facial features, reflecting greater top-down processing. Task 2 behavioral results also suggest that alexithymia does not alter the role or function of emotion concepts in the emotional processing of individuals with high alexithymia (HA); the issue may lie in the activation of emotion concepts.

A more negative N400 amplitude is widely recognized as a neural marker of difficulty in semantic or contextual integration. Furthermore, an enhanced negative N400 indicates greater cognitive resource allocation [78,79]. In Task 1 of the present recognition paradigm, under incongruent contexts, participants with low alexithymia (LA) showed a larger N400 negativity to target faces than participants with high alexithymia (HA), whereas no reliable group difference was observed in congruent contexts. Within our task, this pattern suggests that LA participants allocated more neural resources to processing targets presented in conflicting emotional contexts (e.g., integrating or re-evaluating the context–face relationship during recognition), while HA participants showed reduced modulation by contextual incongruence. These findings suggest that HA participants were less effective at integrating conflicting contextual information. The results from Task 2—where LA participants showed a more negative N400 in the Succeeding Context—imply that they engaged more cognitive resources during top-down processing. Collectively, these findings indicate that, compared with LA participants, HA participants exhibited inefficient allocation of cognitive resources, particularly when resolving conflict and engaging in tasks that demand top-down processing. It is noteworthy that in the Concurrent Context, where bottom-up and top-down processes were relatively balanced and concepts and features carried comparable weights in working memory, HA participants showed the most positive N400 amplitudes. This pattern tentatively points to a potential difficulty in binding features with concepts in HA individuals; however, this interpretation is preliminary and requires confirmation in future research with more specific tests of feature–concept integration.

Overall, these behavioral and ERP results suggest that individuals with high alexithymia have deficits in emotion, potentially due to difficulties in the deliberate activation of emotion concepts.

This study also revealed a noteworthy pattern: even within the low-alexithymia (LA) group, the processing of emotion concepts exhibited significant individual neural differences. Specifically, within the incongruent context of Task 1, the mean N400 amplitude for the LA group was negative but accompanied by considerable variability, where the standard error (SE) exceeded the absolute value of the mean (|M|). A similar pattern of greater variability in the LA group compared to the HA group was also observed in Task 2. This recurring finding indicates that alexithymia is likely not an all-or-nothing discrete category but, rather, a trait dimension that exists on a continuum within the general population. Among so-called “low-alexithymia” individuals, the neural efficiency of the emotion concept system may still vary substantially, which could partly explain why clear between-group differences were not consistently observed at the behavioral level. Future research could employ larger samples to further identify subtypes within this group or investigate how other cognitive traits (e.g., verbal ability, imagination) modulate its neural responses.

Our findings have implications for understanding why individuals with alexithymia fail to form explicit and discrete emotions. According to multiple code theory [80], the progressive transition from the subsymbolic stage (early transient experience) to the symbolic stage (cognitive appraisal and affective expression) enables concepts to serve as tools for distancing from and reflecting initial affective experiences. Concepts are symbolic cognitive functions that facilitate making sense of one’s current situation and sensations, link knowledge and experience to label these experiences as feelings, and communicate these feelings to others [81]. If the activation of emotion concepts was hampered, the transition from the subsymbolic stage to the symbolic stage failed, resulting in the HA individual being confused by ambiguous affective experience rather than forming explicit, discrete emotions.

Our study examined the impacts of conceptual functions on alexithymia, aiming to understand how individuals with alexithymia construct emotions using emotion concepts. Emotional constructivism posits that the foundation of emotion resides in distributed brain networks, rather than in isolated brain areas [82]. Growing evidence supports the constructivist perspective, indicating that emotions are generated by combinations of general psychological processes mapped to large-scale distributed brain networks [83,84,85,86,87]. Critically, the brain regions and networks involved in conceptual processing are deeply intertwined with those supporting emotional cognition. For example, Oosterwijk et al. (2012) demonstrated that simulating emotions, bodily states, or thoughts consistently activates three major networks: the salience network, the default-mode network, and the frontoparietal network [88]. This suggests that emotional processing shares core neural mechanisms with sensory, conceptual, and executive functions. Given this profound neural overlap, investigating populations with impaired emotional conceptualization, such as individuals with alexithymia, provides a crucial test case for the constructivist framework. Neuroimaging studies further reveal that the brain alterations associated with alexithymia are precisely distributed across these sensory, conceptual, and executive networks. Specifically, these alterations involve the prefrontal cortex (executive and conceptual regulation) [89], the anterior cingulate cortex and insula (core regions of the salience network for interoceptive integration) [90,91], and the right amygdala, precuneus, caudate nucleus, hippocampus, and parahippocampal gyrus (regions closely tied to the default mode network, involved in autobiographical memory and situational simulation) [92]. In our study, individuals with high alexithymia showed reduced modulation of the N400 component by contextual incongruence and top-down processes. This finding may relate to brain alterations associated with alexithymia, such as diminished neural responses to negative stimuli in the amygdala and dorsomedial prefrontal cortex, reduced responses to positive stimuli in the right insula and precuneus, and increased dorsal anterior cingulate cortex activity [15]. These patterns may reflect decreased weighting of emotional information and excessive cognitive control during emotional processing in HA individuals. Future research should further investigate these neural mechanisms and their implications for intervention.

It is also important to acknowledge that alexithymia is a multidimensional construct [60,61,62]. For example, factor-analytic work on the TAS-20 consistently supports three related but separable components—Difficulty Identifying Feelings (DIF), Difficulty Describing Feelings (DDF), and Externally Oriented Thinking (EOT)—rather than a single unitary factor. Our analyses focused on overall TAS-20 scores; however, there is growing evidence that these subscales have partly distinct neural correlates. For instance, large-sample VBM work has linked DIF specifically to reduced gray-matter volume in the dorsal anterior cingulate cortex and left temporal regions [93], whereas studies in ALS have found that TAS-20 total and DIF, but not the other subscales, correlate with prefrontal and temporal atrophy [94]. Other work has shown that EOT, but not DIF or DDF, relates to differential activation to masked emotional faces in right supramarginal and inferior frontal regions [95]. EEG-based reviews further suggest that DIF, DDF, and EOT may be associated with partly different patterns of hemispheric asymmetry and large-scale connectivity [96]. Future research with larger samples is therefore needed to determine whether the N400 effects reported here are differentially associated with these specific facets of alexithymia, as well as with alexithymia subtypes derived from further analysis, rather than the TAS-20 total score alone.

## 5. Limitation

We acknowledge that this study is limited by the absence of more precise investigations of brain structure. In addition to the lack of structural neuroimaging data, several further limitations should be noted. First, the sample size was relatively small (*N* = 37), which may reduce statistical power and limit the robustness and generalizability of the present findings. Replication in larger samples is needed. Second, all participants were Chinese, and cultural norms can shape emotion concepts, emotional expression, and alexithymia scores; thus, the results may not readily generalize to other cultural or ethnic groups. Future studies should examine whether similar patterns are observed in more diverse, cross-cultural samples. Third, alexithymia was assessed only with the TAS-20. Although this instrument is widely used and psychometrically sound, reliance on a single self-report questionnaire may not fully capture the complexity of alexithymia. Combining self-report, observer-rated, interview-based, and behavioral measures would provide a more comprehensive assessment in future research.

The study also did not comprehensively examine the effects of other psychological functions, such as perception and executive function, on individuals with alexithymia. Future research should integrate multimodal neuroimaging techniques to further delineate characteristics of brain structure and functional connectivity and systematically incorporate multiple cognitive dimensions, including perception, attention, and executive functions, in order to provide a more comprehensive understanding of the cognitive neural mechanisms underlying alexithymia.

## 6. Conclusions

In this study, behavioral paradigms and ERPs were employed to investigate the effects of alexithymia on neural responses during facial expression processing and to characterize the distinct patterns by which emotion concepts influence emotional processing in individuals with high and low alexithymia. The results demonstrate that individuals with high alexithymia exhibit deficits in emotion perception, potentially due to difficulties with the deliberate activation of emotion concepts. These findings offer a novel perspective on the etiology of alexithymia, suggesting that the crux of alexithymia may lie not only in an inability to describe emotions but also in impaired activation of emotion concepts, leading to a disconnection between conceptual processing and physiological affective experience. This results in an inability to successfully transition from subsymbolic sensory experiences to symbolic conceptual representations. Future research should focus on enhancing emotion concept activation in individuals with alexithymia, and on developing an intervention protocol that facilitates a more seamless and efficient transition from affective experience to expressive articulation.

## Figures and Tables

**Table 1:** Demographics and measurement scores among HA versus LA participants (M ± SD).

Variables	HA	LA	t	*p*	Cronbach’s α
Age	18.57 (0.75)	18.31 (0.60)	0.02	0.984	-
CES-D	5.52 (3.39)	4.93 (3.25)	0.53	0.599	0.85
GAD-7	7.76 (1.22)	7.38 (0.81)	1.16	0.254	0.83
TAS-20	67.60 (5.68)	42.81 (10.29)	8.85	<0.001	0.89

**Table 2:** Bias score and N400 amplitude mean (SE).

Task 1 Bias Score Mean (SE)				
		Context		Group Mean
	Congruent		Incongruent	
HA	0.095 (0.019)		−0.001 (0.028)	0.047 (0.022)
LA	0.065 (0.017)		−0.001 (0.023)	0.032 (0.019)
Context Mean	0.080 (0.020)		−0.001 (0.025)	
Task 2 Bias Score Mean (SE)				
		Context		Group Mean
	Preceding	Concurrent	Succeeding	
HA	0.020 (0.026)	−0.013 (0.022)	−0.112 (0.025)	−0.035 (0.020)
LA	0.036 (0.020)	−0.027 (0.029)	−0.051 (0.035)	−0.014 (0.026)
Context Mean	0.028 (0.023)	−0.020 (0.024)	−0.082 (0.029)	
Task 1 N400 Amplitude Mean (SE)				
		Context		
	Congruent		Incongruent	
HA	1.62 (0.91)		1.62 (0.59)	
LA	0.95 (1.08)		−0.61 (1.04)	
Task 2 N400 Amplitude Mean (SE)				
		Context		
	Preceding	Concurrent	Succeeding	
HA	2.70 (0.97)	3.21 (0.89)	1.02 (0.84)	
LA	1.82 (1.19)	−1.43 (1.04)	−1.73 (0.80)	

## Data Availability

The data presented in this study are available on request from the corresponding author due to privacy protection.

## Abbreviations

ERP	Event-related potentials.
CES-D	Chinese version of the Center for Epidemiological Studies Depression Scale.
GAD-7	Generalized Anxiety Disorder 7-Item Questionnaire.
HA	High alexithymia.
LA	Low alexithymia.
TAS-20	20-Item Toronto Alexithymia Scale.
